# The burden of mental health illnesses in Kerala: a secondary analysis of reported data from 2002 to 2018

**DOI:** 10.1186/s12889-021-12289-0

**Published:** 2021-12-11

**Authors:** Jaison Joseph, D. Hari Sankar, Devaki Nambiar

**Affiliations:** 1 The George Institute for Global Health, 308, Third Floor, Elegance Tower, Plot No. 8, Jasola District Centre, New Delhi, 110025 India; 2 Prasanna School of Public Health, Manipal Academy of Higher Education, Manipal, India; 3 Faculty of Medicine, University of New South Wales, Sydney, Australia

**Keywords:** Mental health, Disability, Surveys, Primary health, Mental health care, Kerala

## Abstract

**Background:**

The burden of mental health in India, as in other Low- and Middle-Income Countries (LMICs), is substantial. Secondary Analysis of survey data provides insight into trends in mental health morbidity over time, while administrative data can indicate corresponding trends in availability of infrastructure and services. We compared data from three national level surveys conducted in India to analyse trends in mental health morbidity and available institutional mechanisms to address mental health needs in Kerala, a south Indian state.

**Methods:**

We compiled data from national and state level population surveys which reported mental health morbidity from 2002 to 2018. We compared the prevalence of mental health illness and disability reported in Kerala with national estimates. We also mapped the most recently available health human resource and infrastructure available in Kerala for mental health care. Basic descriptive statistics were computed for both sets of indicators using Microsoft Excel.

**Results:**

In 2002, Kerala had 194 persons per hundred thousand population with mental retardation and intellectual disability which increased to 300 persons per hundred thousand population in 2018. The number of individuals with mental health illness in the state increased from 272 person per hundred thousand to 400 persons per hundred thousand in the time period of 2002 to 2018. There were 5.53 beds available per ten thousand persons for treatment in Kerala in 2018.

**Conclusion:**

Kerala experienced a rapid rise in mental health morbidity between 2002 and 2018. The most recently reported health human resource and infrastructure availability in the state appears to be inadequate to cater to the requirements of mental health care, even as improvements and upgradations are underway. Service and system design changes will have to be mapped and evaluated over time.

**Supplementary Information:**

The online version contains supplementary material available at 10.1186/s12889-021-12289-0.

## Background

Mental disorders account for the second highest number of Years lived with disability (YLDs) and represent the sixth leading burden in Disability-Adjusted Life Years (DALYs) in the world in 2017 [1]. They also place an enormous economic burden on individuals and societies [2]. A 2016 study on disease burden due to mental disorders by the Indian Council of Medical Research (ICMR) found that one in seven persons in India suffered from mental disorders of varying severity; depression, affecting 45.7 million people, and anxiety disorders, affecting 44.9 million, were the most common [1]. There has been a welcome transformation in the perception of mental illness over the past several decades; with due recognition of the need to safeguard mental health and well-being given in the Sustainable Development Goals (SDGs) [3]. The World Health Organization in its special initiative for mental health (2019–2023) advocated for the inclusion of mental health in the universal health coverage agenda; adding that mental health policies premised on human rights principles should be in place, alongside scaled-up mental health interventions and services at the primary level upwards [4].

Studies show that the prevalence of mental disorders is high among adults in the southern states of India; the burden is concentrated among children and adolescents in the northern states [1, 5]. As per the 2016 Global Disease Burden (GBD) profile of the southern Indian state of Kerala, suicide and violence were among the top 10 causes of death in the 0–14 age group (1.4%), 15–39 age group (24.4%) and 40–69 years (3.9%) [6]. Among the top 15 causes of Years of Life Lost (YLLs) in 2016, suicide ranked third among both male and females. More broadly, the state is facing several challenges due to high morbidity and mortality arising out of the dual burden of communicable and non-communicable diseases, in a population comprised of a growing ageing population, high rates of out migration, and alcohol use [7]. Studies have shown that these factors are associated with mental health disorders [1, 5].

The Kerala state report of the National Mental Health Survey, 2015–16 reported an overall prevalence of any mental disorders of 11.36% [7]. Prevalence of common mental disorders and severe mental disorders were 11 and 0.44% respectively [7]. In 2014 The National Crime Records Bureau (NCRB) estimated that the proportion of persons with high risk of suicidality in Kerala was 2.23% [7]. In 2018 the NCRB reported that the overall incidence rate of suicide per 100,000 persons in Kerala was 23.5, which is double the national rate [8]. The Kerala Disability Census 2015 reported that 8.66% of households in the state had persons with disabilities and 232 persons per 10,000 population suffered from either mental or physical disabilities. Among the total persons with disabilities (7,93,937) 12.72% reported mental illness, 8.68% were persons with intellectual disability, and 0.804% had Cerebral palsy [9].

Studies have examined the point prevalence of mental health disabilities in the state [1, 10–12]. The lack of epidemiological and community-based surveys analysing risk factors and documenting the impact of interventions is a major constraint to understanding mental health challenges in the state [13], particularly over time. While raw survey data could allow some exploration of this, changing diagnostic criteria, differences in assessment and instrumentation and reporting across surveys make it difficult to compare across years and make inferences about trends related to mental health disorders [14]. Yet, there are some surveys that can be used to look at trends, which is what our analysis placed emphasis on.

In terms of policy and programmatic responses to address the aforementioned burdens, superseding the Mental Health Act of 1987, India’s Mental Health Act, 2017 focussed on safeguarding the rights of the people with mental illness, along with access to healthcare and treatment without discrimination from the government [15]. Kerala was among the first in the country to establish a Mental Health Policy in 2003, which was revised in 2013 [7]. It has a district mental health programme functional across all the 14 districts as per the guidelines of National Mental Health Programme (1996) and has shown innovation in policy development and implementation for the care of people with mental health needs [16, 17]. Despite these efforts, the state still has treatment gaps and losses to follow up among those seeking services for mental health disorders in the state [12, 18].

Kerala is not unique here; India’s National Mental Health Survey indicates that mental healthcare services have several underperforming components clubbed with the lack of a comprehensive overall strategy [19]. A strong and effective public health infrastructure can not only to respond to crises but is able to appropriately address the continuum of mental health care needs - preventive, promotive and curative [20, 21]. Globally, in 2014–2016, the median number of beds per 100,000 population requiring mental health inpatient care ranged from below 7 in Low and Middle Income Countries (LMICs) to over 50 in High Income Countries (HICs); the availability of outpatient services was almost 30 times lower in the LMICs as compared to HICs [22]. During this period in India, the availability of beds for mental health in general hospitals per 100,000 population was 0.560, in community residential facilities was 5.178 and in mental hospitals, 1.426 [23]. It is unclear how this availability corresponded to actual burden; this is what our analysis sought to do.

Mental health services rely on the competence and motivation of a skilled workforce [24]. One psychiatrist per 100,000 population is recommended in the Indian scenario by the National Institute of Mental Health and Neuro-Sciences (NIMHANS) [25]. But in 2014, India had only 0.3 psychiatrists per 100,000 population [26]. The median number of psychiatrists in 2017 in the country was only 0.2 compared to global median of 3 per 100,000 population, with similarly low availability of psychologists (0.03 per 100,000), social workers (0.03 per 100,000) and nurses (0.05 per 100,000) available for mental health care [27]. Considering Kerala’s disproportionate burden, it is critical to understand what the availability of these human resources is in the state.

The aim of the current study was to focus on comparable measures available from three large-scale surveys in India to explore a) trends in the prevalence of mental health disorders in Kerala across data sources and b) health infrastructure and human resource available for mental health care reported through national and state surveys.

## Methods

We identified data sources through desk review of large-scale health surveys that reported on mental health between 2002 and 2018. We shortlisted six national level surveys of which three were found suitable for comparison as they had nationally representative samples and have been used by government agencies for formulating policy decisions. We analysed the indicators and definitions that could be used for comparison across the years. While routine data is gathered on a number of indicators, including mental health services, reporting quality has been found to be poor [28]. The data sources used to determine prevalence were the National Sample Survey Office (NSSO) Survey on Disability in its 58th (July – December 2002) and 76th (July – December 2018) rounds and the Census of India 2011. The detailed methodology for each of these studies is mentioned elsewhere [11, 29, 30]; to summarise, the 58th and 76th rounds employed two-stage stratified, random cluster sampling (separately for urban and rural households) with the former round relying on the 2001 Census (excluding geographically remote villages in Jammu & Kashmir, Nagaland, and Andaman and Nicobar Islands) and the latter on the 2011 Census (excluding remote villages in the Andaman and Nicobar Islands). The 2011 Census employed population enumeration in 35 states and Union Territories of the country, in the course of which information on disability was included. More detail on the sampling frame and indicators used in this analysis is provided in supplementary file 1. The 2015–16 National Mental Health Survey report was used to assess the availability of health infrastructure and human resource for mental health care in Kerala; this study design was multi-stage, stratified, random cluster sampling, with random selection of households based on Probability Proportionate to Size (PPS) across stages [7].

Units of measurement reporting mental retardation/intellectual disability and mental illness were standardized to NSS 58th round unit measurements. The NSS 58th round reported number of persons with mental retardation and mental illness per 100,000 population whereas Census 2011 reported number of persons by type of disability and NSS 76th round reported percentage of persons with mental disabilities. The population of Kerala as per Census 2011 population [31] and projected population for 2017 were used for estimations [32]. Human resource and infrastructure data were standardized, and the availability per 10,000 population were assessed, as per World Health Organization (WHO) recommendations [33] .

## Results

### Burden of mental disorders in Kerala

The 2002 NSS 58th round reported 94 persons per 100,000 with intellectual disability; and 105 persons per 100,000 for the mentally ill nationally, whereas in Kerala the figures were 194 and 272 persons per 100,000, respectively. In Kerala, the prevalence of both indicators was higher among males (intellectual disability: 234 per 100,000 males; mental illness: 282 per 100,000 population) as compared to females (intellectual disability: 160 per 100,000 population; and mental illness: 263 per 100,000 population).

Census 2011 data showed that 140 persons per 100,000 population in Kerala had intellectual disability when compared with the Indian average of 124 persons. Similarly, persons with mental illness per 100,000 population was approximately three times higher in Kerala when compared with the national average. NSS 76th round data (from 2018) showed higher burden of intellectual disability and mental illness in Kerala calculated in person per 100,000 when compared with the national average (see Table 1).

**Table 1:** Number of persons with mental retardation and mental illness per 100,000 population from 2002 to 2018

Source of Data	Mental retardation/ Intellectual Disability						Mental illness					
	Kerala			India			Kerala			India		
	Male	Female	Person	Male	Female	Person	Male	Female	Person	Male	Female	Person
NSS 58th 2002	234	160	194	115	72	94	282	263	272	122	86	105
Census 2011	215	168	191	140	108	124	205	185	194	67	52	60
NSS 76th 2018	400	300	300	200	200	200	400	400	400	100	100	100

Source: Compiled by authors from NSS 58th round [29], Census of India 2011 [30], NSS 76th round [11]

### Infrastructure and human resources available for mental health care

As per the most recently available data from 2015 to 16, the availability of mental health care hospitals in the state was less than 0.001 per 10,000 population (see Table 2). The availability of beds for inpatient care was 0.569 per 10,000 population. Only 22% (*N* = 7) of the medical colleges in the state offered psychiatric care. Further, 0.023 sub-district hospitals per 10,000 population provided outpatient/inpatient services for mental health care, whereas none of the primary health care facilities in the reference period could be said to formally deliver care for mental health.

**Table 2:** Availability of Health facilities Available for Mental Health Care in the State

Facility Type	Number	Availability per 10,000 population
Mental hospitals	3	0.001
^a^Medical colleges with psychiatric department	7	0.002
General hospitals with psychiatric unit	18	0.005
Sub district hospital providing OP/IP for mental health	79	0.023
Primary health facilities providing OP for mental health	0	0
Beds available for mental health inpatient services	1962	0.569
Mobile mental health units	22	0.006
Day care centres	43	0.012
Vocational training centres	10	0.003
Sheltered workshops	6	0.002
Long stay homes	146	0.042

^a^ Information pertains to both public and private health care facilities

Source: Health at a glance, DHS Kerala [34], National Mental Health Survey, Kerala report 2015–16

Comparison of Kerala’s human resource for mental health (see Table 3) data with national average and estimated requirement showed that the state currently possess 400 psychiatrists, representing a coverage of 0.12 per 10,000 population which is better than national average (0.067 per 10,000 population), but falls short of recommended coverage of the mental health workforce in India [35]. This pattern is consistent with data on other human resources for mental health like clinical psychologists, but the availability of psychiatric social workers in Kerala was less than national and very low when compared to the estimated requirement.

**Table 3:** Human Resources available for Mental Health Care in Kerala and India

Type	Kerala		India		Requirement per 10,000 population
	Number of Health Workers	Availability per 10,000 population	Number of Health Workers	Availability per 10,000 population	
Psychiatrist	400	0.12	9000	0.067	3
Clinical psychologist	211	0.06	1000	0.007	3
Psychiatric Social workers	15	0.004	1000	0.007	3
Rehabilitation workers and special education teachers	3429	1	2649	0.020	3
Nurses with Diploma in Psychiatric Nursing	0	0	2000	0.015	3

Estimations for mental health workforce by Garg et. al for India based on Global Mental Health Norms [35]

Source: National Mental Health Survey, Kerala report 2015–16

## Discussion

We examined summary trends in prevalence of mental health conditions from 2002 to 2018 reported in nationally representative surveys and the latest status of mental health service delivery infrastructure and human resources coverage in the state of Kerala. Overall, we found that burden was greater in Kerala as compared to national averages and has been steadily growing. We also found that the availability of infrastructure and health workers falls short of globally recommended thresholds.

A third of patients seeking care in community based or psychiatric hospital setting have been reported to experience internalised stigma [36]. Even accounting for under-reporting in surveys assessing mental disability, prevalence is increasing. Indeed, actual burden may be higher in Kerala, but is the level of health literacy and health seeking in the state, in a larger context where emphasis is being placed on reporting on this issue. This is a clear area for further study. Studies have attributed the rise of depression in Kerala to a shift in family patterns from joint to nuclear families and the associated rise in loneliness and abandonment of the elderly, rising divorce rates, unemployment, gender inequalities, economic migration to countries in the Middle East/Gulf region (with wives staying behind in Kerala), overconsumption, pressure on students, alcoholism, and tensions associated with modern living [17, 37]. National Crime Records Bureau (NCRB) data from 2016 report that family problems, illness, alcohol/ drug abuse and bankruptcy are also major drivers of suicide in India [38]. Clearly, more study is needed in this domain.

Having been the first Indian state to formulate a mental health policy (in 2003), Kerala has been expanding services in order to offer screening and management of common mental health conditions at the primary health care level [39]. In 1999 the District Mental Health Programme (DMHP), and Direct Intervention System for Health Awareness (DISHA) released in 2013 were designed to strategize around and implement mental health initiatives by expanding the district mental health program and integrating it with the private sector [40]. Alongside this, strong referral pathways have to be established to provide more specialised care for mental health [41]. These programmatic aspects need to be assessed and evaluated.

Studies have indicated a lack of mental health service provision in primary health centres in the state [40, 42]. This is now expected to change given the reforms brought about under the aegis of Kerala’s Aardram flagship initiative, which established depression screening clinics named *Ashwas* (which roughly translates into “assurance”) (initially) in nearly 170 Family Health Centres (FHCs) across the state. *Ashwas* doctors and staff nurses are trained in psycho-social counselling and clinical guidelines, and are directed to manage screening for depression and treatment at FHCs, while trained field health workers are to identify cases in defined vulnerable and high risk groups for referral to FHCs [43]. The degree to which this program can improve diagnosis, treatment and care of those with mental health conditions is a critical area for further monitoring and research. The DHMP and *Ashwas* clinics are joined by the private sector in providing services in the face of continued calls for improved accessibility, availability, and quality of mental health services and family-support services for this target population [40, 44, 45]. This is another area of further study.

Delivery of services for mental health depends on the availability of human resources for providing such services. India has a long way to go in this area: a 2011 WHO report found that for every million people in India, there are just three psychiatrists, and even fewer psychologists, 18 times fewer than the Commonwealth norm of 5.6 psychiatrists per 100,000 people [46]. In Kerala, the number of psychiatrists available per thousand population was higher than the Indian average, however there were relatively fewer clinical psychologist, psychiatric social workers and no psychiatric nurses available in the state. The shortage of mental health professionals is especially acute in the public sector [42]. An assessment conducted in 2013 found that district level shortages abound; 75% of psychiatrist positions were found to be vacant in Malappuram and Palakkad districts [47]. This has been tied to medical education; Roy and Rasheed found a deficiency in the number of psychiatry post graduate seats, attributing this to the inadequate number of psychiatrists in the country [25]. These shortages can be addressed with appropriate training for health care professionals at the primary level [48] as well as non-specialist health-care providers [12].

The toll of COVID-19 on mental health is well established [49]: globally, around three fourths of COVID- affected persons have been diagnosed with posttraumatic stress disorders (PTSD) along with depressive and anxiety disorders [50]. Studies have found that health providers are facing stress, anxiety, depressive symptoms, insomnia, denial, anger and fear due to social isolation [51, 52]. People in quarantine have reportedly experienced boredom, loneliness, anger, anxiety and guilt about the effect of contagion and stigma on family and friends [52, 53]. A recent study found that during the COVID-19 pandemic in Kerala, depression was a major problem faced by people under home quarantine (75.2%) followed by stigma (69.5%) and anxiety (69.4%) [54]. In response, the state adopted inter and intra-departmental coordination to ensure continuity of services and access to additional support like medications and rations – in part to mitigate these challenges [52]. Clearly more is needed: COVID has starkly revealed that both Kerala and India are indicative of the phenomenon common in Low and Middle Income Countries (LMICs), where despite substantial and growing burdens, support for mental health and psychological care is vitiated by scarcity of resources and lack of adequate information in the population [41]. Research and programmatic support are required to strengthen these responses.

### Limitations

Despite the similarity of domains analysed, the scale of measurement differed for most of the surveys. Some reported in percentages, others in proportions and some in absolute numbers, which raised issues of comparability across the years. The focus of mental health surveys has also varied over the years and now perception of mental illness has changed resulting in change of operational definitions, again limiting comparability over the years. Ideally, secondary data would be triangulated across multiple data sources to improve quality and ensure reliability [55]. This is of course difficult to do with survey data, which is of long periodicity typically, and tends not to afford such comparison or triangulation. Often, routine health data can be used for this purpose. In our case, however, routinely collected departmental data on mental health illness in the state was not available in the public domain. A report by NITI Aayog, a premier think tank of the Indian government, has also raised concern that routinely collected data in our public health system has quality issues (like incompleteness and lack of validation) [56]. Another limitation in our analysis was our inability to look at population subgroups facing greater burdens of mental illness. Future research should draw from across sources to carry out more precise and triangulated analyses, adjusting for age, sex, as well as other dimensions of inequality, such as socio-economic status, occupation, and more.

## Conclusion

There appears to be a substantial increase in mental health illness over the 16-year study period in the state of Kerala. The current health infrastructure in the public sector of the state has so far been inadequate to meet the current burden of the problem and to ensure universal health coverage to its population. Kerala’s increased emphasis on mental health as per recent reforms is a welcome change and should be monitored using standardised definitions in both routine health information systems and large population-based surveys, enabling comparability over time.

## Supplementary Information

**Additional file 1.** Methodology and Indicators for Analysis.

## Data Availability

All data collected and analysed during this study are sourced from data sources available in the public domain and the links to these sources are given below. The data management and analysis plan are detailed in method section and the supplementary information files. These sources are open to access as on 28th October 202. Data source 1: Disabled persons in India – NSSO 58th round available from http://www.icssrdataservice.in/datarepository/index.php/catalog/69/download/877. Data source 2: Disabled persons in India a statistical profile 2016 available from http://mospi.nic.in/sites/default/files/publication_reports/Disabled_persons_in_India_2016.pdf. Data source 3: Persons with disabilities in India- NSSO 76th round available from http://mospi.nic.in/sites/default/files/publication_reports/Report_583_Final_0.pdf. Data source 4: Health at a glance 2018 available from https://dhs.kerala.gov.in/wp-content/uploads/2020/03/health_25022019.pdf. Data source 5: National mental health survey of India 2015–16 Kerala report available from http://indianmhs.nimhans.ac.in/Docs/statereports/Kerala-NMHS-Report.pdf.

## Abbreviations

DALYs: Disability Adjusted Life Years
DMHP: District Mental Health Programme
DISHA: Direct Intervention System for Health Awareness
FSUs: Sample First Stage Units
ICMR: Indian Council of Medical Research
LMICs: Low- And Middle-Income Countries
NCRB: National Crime Records Bureau
NIMHANS: National Institute of Mental Health and Neurosciences
NITI: National Institution for Transforming India
NMHS: National Mental Health Survey
NSS: National Sample Survey
NSSO: National Sample Survey Office
ORGCS: Office of Registrar General and Census Commissioner
PTSD: Posttraumatic Stress Disorders
PWD Act: Persons with Disabilities Act
SDG: Sustainable Development Goal
SDH: Sub District Hospital
SDI: Socio-Demographic Index
WHO: World Health Organization
YLDs: Years Lived with Disability
YLLs: Years of Life Lost
