# Supplementary material for: The burden of mental health illnesses in Kerala: a secondary analysis of reported data from 2002 to 2018
Source: BMC Public Health. 2021 Dec 11;21:2264. doi: 10.1186/s12889-021-12289-0 (PMC8665697; doi:10.1186/s12889-021-12289-0)
Supplement: Supplementary file 1 — Additional file 1. Methodology and Indicators for Analysis. [file 12889_2021_12289_MOESM1_ESM.pdf]

Supplementary File -1

Methodology and Indicators for Analysis

The National Sample Survey (NSS) in its 58th round (July-December 2002) included mental disability in addition to the three physical disabilities visual, communication (i.e. Hearing and or speech) and locomotor that was covered during the previous rounds. NSS 58th round surveyed 45,571 rural and 24,731 urban households respectively from 4,637 villages and 3,354 urban blocks enumerating 49,300 rural and 26,679 urban disabled persons (National Sample Survey Organisation Ministry of Statistics and Programme Implementation Government of India, 2003).

The 2011 Census collated information on eight types of disability (disability in seeing, in hearing, in speech, in movement, in mental retardation, in mental illness, any other and multiple disability) by covering all types of disabilities listed in the Persons with Disabilities Act, 1995 and National Trust Act, 1999. Information on disability was collected during the Population Enumeration phase of Census 2011 through 'Household Schedule' conducted by the Office of Registrar General and Census Commissioner(ORGCS), the 15th Indian Census sampled 7,935 urban and 640,867 rural households in 35 states and Union Territories (Social Statistics Division, Ministry of Statistics and Programme Implementation, 2016).

Further, the 76th NSS round (July – December 2018) classified persons with disabilities as per the classification used in the Rights of Persons with Disabilities Act (PWD Act) 2016 with the objective to estimate indicators of incidence and prevalence of disability, cause of disability, age at onset of disability, facilities available to the persons with disability, difficulties faced by persons with disability in accessing/using public building and public transport, arrangement of regular care giver, out of pocket expenses relating to disability, etc. The NSS 76th round comprised 8,992 sample First Stage Units (FSUs) (5,378 FSUs in rural areas and 3,614 FSUs in urban areas) covering 1,18,152 households (81,004 in rural areas and 37,148 in urban areas) and enumerating 5,76,569 persons (4,02,589 in rural areas and 1,73,980 in urban areas). The total number of persons with disability surveyed was 1,06,894 (74,946 in rural areas and 31,948 in urban areas) (Ministry of Statistics and Programme Implementation, Government of India, 2018).

The National Mental Health Survey, Kerala through a representative sampling frame assessed the availability of existing services and resources relevant to mental health in the state. The following domains were assessed to identify the services for mental health: 1) General information about the state 2) General health resources in the state 3) Existing mental health systems and resources in the state 4) Mental health policy & action plan 5) State authorities responsible for mental health activities 6) Legislation and implementation related to mental health. A total of 1223 households, with 3149 eligible individuals, were contacted and achieved 2479 adults from 926 households (National Institute of Mental Health and Neurosciences and Institute of Mental and Neuro Science, 2016).

References

Ministry of Statistics and Programme Implementation, Government of India. (2018). *Persons with Disabilities in India*.

National Institute of Mental Health and Neurosciences and Institute of Mental and Neuro Science. (2016). *National Mental Health Survey of India 2015-16, Kerala*.

National Sample Survey Organisation Ministry of Statistics and Programme Implementation Government of India. (2003). *Disabled Persons in India, NSS 58th Round*. Retrieved from http://www.icssrdataservice.in/datarepository/index.php/catalog/69/download/877

Social Statistics Division, Ministry of Statistics and Programme Implementation. (2016). *Disabled persons in India a Statistical Profile*.
